# Detection of GD2-positive cells in bone marrow samples and survival of patients with localised neuroblastoma

**DOI:** 10.1038/sj.bjc.6604179

**Published:** 2008-01-08

**Authors:** M V Corrias, S Parodi, R Haupt, L Lacitignola, F Negri, A R Sementa, D Dau, F Scuderi, B Carlini, M Bianchi, F Casale, L Faulkner, A Garaventa

**Affiliations:** 1 Department of Experimental and Laboratory Medicine, Laboratory of Oncology, Gaslini Institute, Largo Gaslini, 5, Genoa 16147, Italy; 2 Department of Experimental and Laboratory Medicine, Epidemiology and Biostatistics Section, Scientific Directorate, Gaslini Institute, Largo Gaslini, 5, Genoa 16147, Italy; 3 Meyer Children's Hospital, Via Luca Giordano 13, Florence 50132, Italy; 4 Service of Pathology, Gaslini Institute, Largo Gaslini, 5, Genoa 16147, Italy; 5 Department of Hematology–Oncology, Gaslini Institute, Largo Gaslini, 5, Genoa 16147, Italy; 6 Pediatric Oncology, Ospedale infantile Regina Margherita, Piazza Polonia 94, Torino 10126, Italy; 7 Department of Pediatrics, II University of Naples, Via Sant'Andrea 4, Naples 80138, Italy

**Keywords:** neuroblastoma, detection, GD2, immunocytochemistry, survival

## Abstract

The impact of bone marrow (BM) GD2-positive cells on survival has been evaluated in 145 Italian children with localised neuroblastoma (NB) evaluated at diagnosis by anti-GD2 immunocytochemistry. Nineteen of these (13.1%) were found to be BM GD2-positive, with the number of positive cells ranging between 1 and 155 out of 1 × 10^6^ total cells analysed. Seven/19 (38.8%) GD2-positive *vs* 12/126 (9.5%) GD2-negative patients relapsed. The 5-year event-free survival (EFS) and overall survival of the GD2-positive patients was significantly worse than that of the GD2-negative ones (62.2 *vs* 89.9%, *P*<0.001; and 74.9 *vs* 95.9%, *P*=0.005, respectively). GD2 positivity was not associated to other known risk factors, and in particular to *Myc-N* amplification and 1p deletion. Among *Myc-N*-negative patients, the EFS of those negative for both GD2 and 1p deletion was significantly better than in children positive for either one of these two markers (EFS=96.9 *vs* 66.0%, *P*<0.001). In conclusion, GD2 positivity may represent a prognostic marker for patients with non-metastatic NB without *Myc-N* amplification, and its combination with genetic alterations might help identifying patients that require a more careful follow-up.

Neuroblastoma (NB) is the most common extracranial solid malignancy of childhood (Henry *et al*, 2005). Staging, clinical management and prognosis mainly depend on the presence/absence of bone marrow (BM) and skeletal involvement (Brodeur *et al*, 1993). Almost 50% of NB patients present at diagnosis with localised disease, that is, they do not have evidence of BM metastases, as assessed by morphological examination of both marrow smears and trephine biopsies, nor other distant localisations investigated by ^123^I-MIBG scintigraphy.

Usually patients with localised disease are treated by surgery alone (stage 1 and 2) or by standard-dose chemotherapy followed by surgery (stage 3), unless amplification of the *Myc-N* proto-oncogene is detected in their tumour cells (Rubie *et al*, 1997b; Haase *et al*, 1999; Simon *et al*, 2004; Henry *et al*, 2005; Maris, 2005), which requires a more aggressive chemotherapeutic regimen. Event-free (EFS) and overall (OS) survival of the patients with localised disease without *Myc-N* amplification are good (95% for stage 1, 86% stage 2 and 65% for stage 3 patients; Cotterill *et al*, 2000), but a small percentage of them relapse and may die of disease.

Genetic abnormalities at chromosome 1p (Rubie *et al*, 1997a), 3p, 11q (Spitz *et al*, 2003; Attiyeh *et al*, 2005; Simon *et al*, 2006) and 17q (Brinkschmidt *et al*, 2001), as well as biochemical (Simon *et al*, 2003), histological (Perez *et al*, 2000; Navarro *et al*, 2006; Sano *et al*, 2006), and biological factors (Christiansen *et al*, 1995; Cheung *et al*, 1997; Kramer *et al*, 1997; Perez *et al*, 2000; Ladenstein *et al*, 2001; Mora *et al*, 2001; Krams *et al*, 2003; Riley *et al*, 2004; Haber *et al*, 2006; Spitz *et al*, 2006), do not seem to have the same relevance in patients with localised NB as they do in patients with metastatic disease. Gene expression profiling and GCH studies have suggested specific favourable and unfavourable NB signatures (Takita *et al*, 2004; Ohira *et al*, 2005; Vandesompele *et al*, 2005), but presently a widespread identification of patients at risk of relapse by these techniques cannot be envisaged. Therefore, an independent, easily applicable, prognostic marker able to identify patients that would benefit from a more careful follow-up is currently lacking.

In a previous study, aimed to assess the diagnostic and prognostic role of different techniques detection of NB tumour cells in peripheral blood and BM, we observed that in patients with localised NB the GD2 positivity in BM was negatively associated with survival (Corrias *et al*, 2004). However, this finding was based on a small sample size with a relatively short follow-up. The aim of the present investigation was to evaluate the impact of BM GD2 positivity, evaluated at diagnosis by anti-GD2 immunocytochemistry (IC), and its combined effect with other known risk factors on survival of a larger cohort of patients with localised NB.

## MATERIALS AND METHODS

### Patients

One hundred and forty-five consecutive NB patients, diagnosed with localised disease (stages 1–3) according to INSS criteria (Brodeur *et al*, 1993) at 20 Italian paediatric oncology centres between January 1997 and June 2003, with available information on BM GD2 status at diagnosis, were included in the study. Disease staging (Brodeur *et al*, 1993) at diagnosis, including appropriate imaging, ^123^I MIBG scintigraphy and BM evaluation, was made at the referring oncology centre and centrally reviewed at the Gaslini Institute.

Therapeutic approach for stage 1–2 patients included only surgery followed by a complete re-evaluation with appropriate imaging 1 month later. Stage 3 patients also received chemotherapy according to national or international protocols. Stage 3 and 2 *Myc-N*-amplified patients received high-dose chemotherapy, myeloablative therapy with haematopoietic stem cell rescue and local radiotherapy. In case of relapse, a complete restaging, including imaging and BM evaluation, was performed.

For each patient, demographic, clinical and follow-up data, together with information on biological characteristics and other prognostic risk factors as serum LDH, NSE and ferritin, tumour *Myc-N* amplification and 1p status (Ambros *et al*, 2003), are summarised in Table 1. Data were retrieved from the Italian Neuroblastoma Registry (INBR) that collects information on clinical and biological characteristics of patients at diagnosis as treatment; follow-up is sought during protocol administration and then at least yearly after treatment discontinuation (Conte *et al*, 2006). Pathology data regarding the primary tumour were not considered for this study, since information according to the criteria proposed by the International Neuroblastoma Pathology Committee (INPC) was not available for patients diagnosed before 2003.

### BM analysis

In general, BM aspirations and bone trephine biopsies at both iliac crests were performed under general anaesthesia during surgical procedure on the primary tumour or during ‘*ad hoc*’ sedation. For morphological analysis, three May Grünwald–Giemsa-stained slides from each site were examined by an experienced cytomorphologist and centrally reviewed. Trephine biopsies were obtained by a Jamshidi needle, and only biopsies containing at least 5 mm^3^ of tissue were considered adequate for evaluation. At least 30 high-resolution fields of haematoxylin–eosin-stained sections were evaluated by an experienced pathologist and centrally reviewed.

For the purpose of this study, GD2-IC was centrally performed at the Italian NB reference laboratory, as previously described (Corrias *et al*, 2004). Briefly, six cytospins, each containing 5 × 10^5^ mononuclear cells, were fixed in cold acetone and incubated with the 3F8 anti-GD_2_ mAb (kindly donated by Dr Nai-Kong Cheung, Memorial Sloan Kettering Cancer Center, New York, NY, USA). After washing, slides were incubated with a biotinylated anti-mouse antibody and developed with an avidin–alkaline phosphatase conjugate (DAKO, Copenhagen, Denmark). Enumeration of GD2-positive cells was based on both morphological and immunological criteria, according to standardised conditions (Swerts *et al*, 2005). Namely, the presence of round nuclei larger than that of small lymphocytes, granular chromatic structure and scarce amount of cytoplasm were considered positive morphological criteria; strong red staining localised to the entire cell membrane and cytoplasm was the positive immunological criterion. Bone marrow samples were considered positive if at least three positive tumour cells were detected out of the 3 × 10^6^ cells analysed.

The study was approved by the Institutions' Ethical Committees. All analyses were performed after informed consent was given from the patients themselves or their legal guardians, according to the Helsinki declaration.

### Statistical analysis

Descriptive statistics were reported as percentages for categorical variables. For continuous and counting data, medians with interquartile range (IQR) were used due to the non-normal distribution of the observations and to reduce the effect of outliers. Patients were stratified according to their BM GD2 status and comparisons of frequency data were performed by means of the *χ*^2^-test or the Fisher's exact test, when appropriate. The Wilcoxon Mann–Whitney test was used to compare median values, while the Spearman *ρ* coefficient was used to assess correlation between variables. Event-free survival and OS analyses were performed according to the Kaplan–Meier method and compared by the log-rank test. A *P*-value <0.05 was considered as statistically significant. Analyses were performed using Stata for Windows statistical package (release 7.0; Stata Corporation, College Station, TX, USA).

## RESULTS

During the study period, 145 patients diagnosed with localised NB and registered in the INBR had their BM aspirates analysed at diagnosis by anti-GD2-IC. Patients included in the study were similar for age, sex, stage, *Myc-N* status and survival to the children with localised NB without information on GD2 status at diagnosis (see Supplementary data).

**Figure 1:**
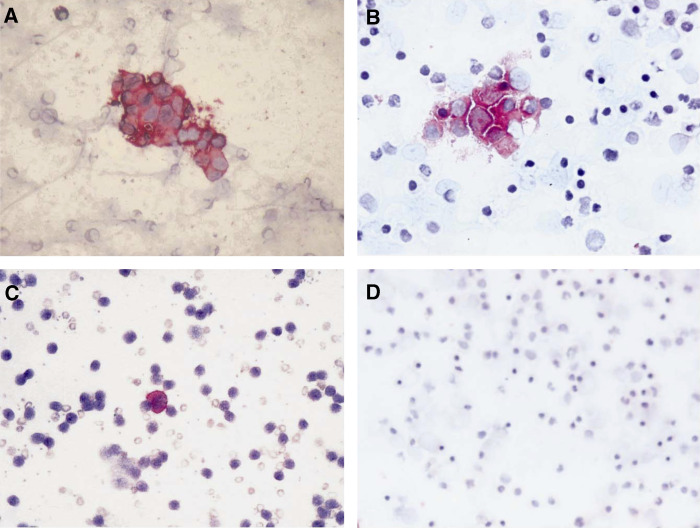
Cytospin of BM aspirates fixed in acetone and immunologically stained with anti-GD2 antibody. (**A**, **B**) Rosettes of NB cells stained in red, from patients F/18 and F/8, respectively; (**C**) a single NB cell stained in red from patient M/73; (**D**) a completely negative aspirate. Magnification is × 40.

Demographic, clinical, biochemical and genetic features of the 145 study patients stratified according to BM GD2-IC status are reported in Table 1. In more detail, 126 patients (86.9%) were GD2 negative and 19 (13.1%) were GD2 positive. Among the 19 GD2-positive patients (Table 2), the number of positive cells ranged between 1 and 155 (median=3; IQR 2-20) out of 10^6^ total cells examined. It is to be noted that of the 11 patients with less than five GD2-positive cells/10^6^ total cells, seven were also evaluated by RT-PCR and all but one were found to be positive for at least one NB molecular marker (data not shown). Three examples of GD2-positive samples are shown in Figure 1. As reported in Table 1, no association was found between GD2 status and each of the other risk factors considered.

**Figure 2:**
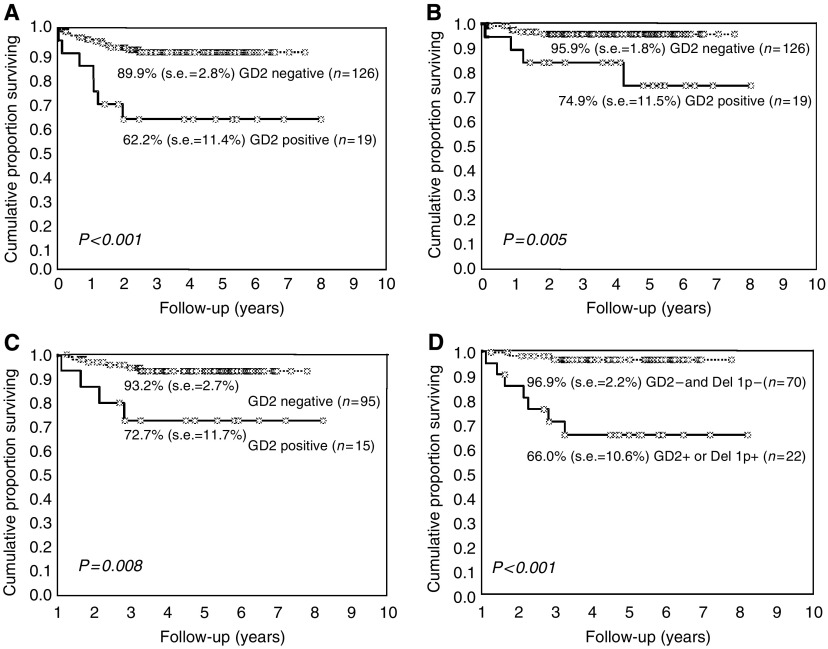
(**A**) Event-free survival of patients with localised NB stratified by BM GD2-IC status. (**B**) Overall survival of patients with localised NB stratified by GD2-IC status. (**C**) Event-free survival of patients with normal *Myc-N* status stratified by GD2-IC status. (**D**) Event-free survival of patients with normal *Myc-N* status stratified by GD2-IC and chromosome 1p status.

Seven (36.8%) of the 19 GD2-positive patients relapsed, all locally, and four of them died due to local disease progression (Table 2). The 5-year EFS and OS of this group were 62.2 and 74.9%, respectively (Table 1; Figure 2A and B). In these patients, an inverse correlation between the number of GD2-positive cells and the time to relapse was observed (*ρ*= −0.786, *P*=0.036). Conversely, among the 126 BM GD2-negative patients, only 12 (9.5%) relapsed, 10 locally and two with metastatic disease, and five subsequently died (Table 2) with a 5-year EFS of 89.9% and an OS of 95.9% (Table 1; Figure 2A and B). Differences in EFS and OS between the two groups were significant (*P*<0.001 and *P*=0.005, respectively; Table 1). If, as in our previous study, the five cells/10^6^ cut off was used to discriminate positive BM samples, differences in survival remained highly significant (data not shown). Table 3 further reports on the 5-year EFS analyses on the entire cohort also considering other clinical and biological risk factors. A statistically significant worse effect was observed for unresectable disease (i.e., stage 3) (EFS=76.7%) *vs* resectable disease (i.e., stage 1–2) (EFS=89.8%; *P*=0.033), high LDH levels (72.1 *vs* 89.5%, *P*=0.010), *Myc-N* amplification (EFS=57.1 *vs* 90.4%, *P*<0.001) and chromosome 1p status (EFS=45.8% for deletion, 83% for imbalance and 92.7% for normal, *P*<0.001). The negative effect of 1p deletion remained even if 1p imbalance was pooled with the not deleted cases (data not shown); for this reason, in subsequent analyses patients with 1p imbalance were grouped with the not deleted ones.

Since *Myc-N* status directs patients towards either standard or high-risk treatment, survival analysis was repeated in patients with and without *Myc-N* amplification. In *Myc-N*-amplified patients, GD2 negativity was associated with a better survival (EFS 66 *vs* 0%), but the difference was not significant (*P*=0.073), likely due to the small number of cases. However, in *Myc-N* non-amplified patients, the BM GD2 negativity was associated with a significantly better outcome (EFS=93.2 *vs* 72.7%, *P*=0.008; Figure 2C). In the same *Myc-N*-negative patients, the combined prognostic role of GD2 and 1p status was also assessed. Patients negative for both markers had a 96.9% EFS, which was significantly better than that observed among children positive for at least one marker (EFS=66.0%; *P*<0.001; Figure 2D). In the same group of *Myc-N*-negative patients, the OS showed a similar pattern (98% for the GD2-negative subjects *vs* 83% for the GD2-positive subjects). Finally the OS of patients positive for either GD2 or *Myc-N* was worse than that of patients negative for both the markers (83.1 *vs* 100%), but the very low number of observed deaths prevents one to draw definitive conclusions.

Finally, among BM GD2-IC-positive patients, a worse survival was found in children with ⩾20 GD2-positive cells/10^6^ total cells, corresponding to the fourth quartile (EFS=77.1 *vs* 20.0%, *P*=0.002). A similar result was also observed after excluding the two patients with *Myc-N* amplification (data not shown).

Because of the small sample size (19 BM GD2-positive patients) and low number of events, results from a multivariate analysis should be taken with caution and are herewith reported only as Supplementary data. In this analysis, the GD2-IC status seemed to maintain its prognostic role independently from both *Myc-N* amplification and 1p deletion. It is of note that conversely *Myc-N* amplification and 1p deletion were indeed correlated, being 72.7% of *Myc-N* amplified patients also deleted of 1P.

## DISCUSSION

This study has confirmed our previous observation (Corrias *et al*, 2004) of a negative impact of BM GD2-positive cell infiltration on survival of patients with localised NB. In particular, in this larger cohort of patients with a longer follow-up, we have shown that this effect is not due to the association with other risk factors as *Myc-N* amplification and 1p deletion.

Other reports have previously documented that tumour cells may be detected by IC and/or RT-PCR in BM of children with localised NB (Moss *et al*, 1991; Cheung *et al*, 1998; Trager *et al*, 2003; Corrias *et al*, 2004; Simon *et al*, 2004; Ifversen *et al*, 2005; Russell *et al*, 2005; Swerts *et al*, 2006). However, the limited number of patients analysed and/or the short follow-up of those studies did not allow one to draw definitive conclusions on the prognostic impact of this detection. In addition, a large multi-centre European study (Navarro *et al*, 2006) and several other national studies (Christiansen *et al*, 1995; Cheung *et al*, 1997; Ladenstein *et al*, 2001; Simon *et al*, 2003; Sano *et al*, 2006; Spitz *et al*, 2006) have indicated that stage and *Myc-N* status were the only independent risk factors for children with localised NB. However, *Myc-N* amplification is a relatively rare event, occurring, as in our series, in about 10% of localised NB patients (Haase *et al*, 1999; Perez *et al*, 2000; Henry *et al*, 2005; Maris, 2005) and is thus inadequate to identify all the patients who will eventually relapse.

A negative prognostic role of unfavourable histology (Perez *et al*, 2000), of 11q LOH (Attiyeh *et al*, 2005; Simon *et al*, 2006), and of a specific GCH profile (Schleiermacher *et al*, 2007), was also previously reported in patients with localised disease. This effect was independent from *Myc-N* status. Unfortunately, homogeneous information on tumour histology (i.e., INPC classification) was not available in our registry because of the long recruitment period covered by this study. Moreover, our patients were not screened for genetic abnormalities other than *Myc-N* amplification and 1p deletion. Even if genetic screening could have been more precise, our observation of a negative prognostic role of BM GD2-IC positivity suggests that also this technique, which has become accurate, thanks to recent standardisation (Swerts *et al*, 2005), might be of interest in future studies.

The low relapse rate observed in our cohort is similar to that expected in patients with localised NB, and indicates that staging was correct. The fact that presence of GD2-positive cells in the BM correlated with a higher tendency of relapse at the primary site, instead that in the BM, is surprising. However, also Perez *et al* (2000) did not observe further BM involvement in eight stage 1–2 patients found positive by BM immunocytology. Thus, it is conceivable that the very few GD2-positive cells, detected by IC at diagnosis, were unable to survive and actively proliferate in the BM. Whether this inability to invade the marrow compartment was related to the NB cells themselves or to the presence/absence of factors in the BM microenvironment (Fidler, 2003) remains to be determined in future studies.

Our observation that higher number of GD2-positive cells correlated with poorest survival and with a shorter time to relapse is similar to that previously reported by Moss *et al* (1991). Even if this finding was based on a very small sample size (i.e., seven relapses in GD2-positive patients), it suggests that quantification by IC might add useful clinical information and that BM GD2-IC positivity reflects a biological feature of the neuroblasts that can affect the disease course.

In our study, survival analysis on other prognostic factors confirmed the negative role of *Myc-N* amplification, high LDH serum levels and 1p deletion. Only a multivariable analysis based on a larger series will be able to assess the combined effect of BM GD2 positivity and the other known major risk factors. However, our data suggest that in patients with localised disease without *Myc-N* amplification, the combination of BM GD2-IC and 1p status might help individuating those at risk of relapse.

In conclusion, we believe that BM GD2-IC analysis might have the potential to discriminate different risk groups within localised NB patients. In fact, GD2-IC seems to provide additional information on biological features and has the advantage of generating quantitative data. We neither recommend that BM GD2-IC status be used for staging purposes, nor the patient be shifted to a more intensive treatment because not all the GD2 positive patients relapsed. However, in future multi-national studies, patients with localised disease should be evaluated by GD2-IC. In fact, in the presence of BM GD2-positive cells, especially if combined with chromosome 1p deletion, patients should be closely followed, and in case of relapse, they should be treated more aggressively.

## Figures and Tables

**Table 1:** Demographic, clinical, biochemical and genetic features of the 145 patients with localised NB stratified by GD2 status

	**GD2 negative (*n*=126)**		**GD2 positive (*n*=19)**			**Total (*n*=145)**	
	***n***	**%**	***n***	**%**	***P***	***n***	**%**
*Gender*					0.059		
Male	69	54.8	6	31.6		75	51.7
Female	57	45.2	13	68.4		70	48.3
							
*Age*					0.932		
<12 months	61	48.4	9	47.4		70	48.3
⩾12 months	65	51.6	10	52.6		75	51.7
							
*Primary site*					0.923		
Neck	7	5.6	0	0.0		7	4.8
Thorax	29	23.0	4	21.1		33	22.8
Abdomen, adrenal	44	34.9	7	36.8		51	35.2
Abdomen, other sites	46	36.5	8	42.1		54	37.2
							
*INSS stage*					0.315		
Stage 1	59	46.8	7	36.8		66	45.5
Stage 2	35	27.8	4	21.1		39	26.9
Stage 3	32	25.4	8	42.1		40	27.6
							
*NSE (78 tested)*					0.660		
<100 ng ml^−1^	57	89.1	12	85.7		69	88.5
⩾100 ng ml^−1^	7	10.9	2	14.3		9	11.5
							
*LDH (123 tested)*					0.999		
<1000 IU ml^−1^	86	81.9	15	83.3		101	82.1
⩾1000 IU ml^−1^	19	18.1	3	16.7		22	17.9
							
*Ferritin (95 tested)*					0.999		
<150 ng ml^−1^	63	77.8	11	78.6		74	77.9
⩾150 ng ml^−1^	18	22.2	3	21.4		21	22.1
							
*Myc-N (124 tested)*					0.999		
Not amplified	95	88.8	15	88.2		110	88.7
Amplified	12	11.2	2	11.8		14	11.3
							
*1p36 (105 tested)*					0.999		
Not deleted	60	67.4	11	68.8		71	67.6
Deleted	14	15.7	2	12.5		16	15.2
Imbalance	15	16.9	3	18.8		18	17.1
							
5-year EFS	126	89.9	19	62.2	<0.001		
5-year OS	126	95.9	19	74.9	0.005		

EFS=event-free survival; NB=neuroblastoma; OS=overall survival.

**Table 2:** Features of the GD2-positive patients and of the GD2-negative patients who relapsed

**Stage**	**Sex/age (months)**	**GD2+ cells/10^6^ total**	**Tumour site**	**LDH** **(IU ml^−1^)**	***Myc-N***	**1p**	**Relapse (months)**	**Type of relapse**	**Follow-up^a^**
1	M/45	1.3	Thorax	650	Normal	Normal	—	—	CR
1	F/2	1.0	Abdomen	475	Normal	Normal	—	—	CR
1	M/25	2.2	Pelvis	523	Normal	Normal	—	—	CR
1	F/93	1.6	Thorax	601	Normal	Normal	—	—	CR
1	F/2	3.0	Abdomen	411	ND	ND	—	—	CR
1	M/2	10.0	Abdomen	494	Normal	Normal	—	—	CR
1	M/6	39.3	Abdomen	1961	Amplified	Deleted	1.8	Local	DPD
									
1	F/10	Negative	Abdomen	1693	Amplified	Deleted	1.4	Local+Met	DPD
1	F/25	Negative	Thorax	617	ND	ND	18.0	Local	AWD
1	F/1	Negative	Abdomen	NE	Normal	Normal	10.0	Local+Met	AWD
									
2	F/59	8.0	Abdomen	339	Normal	ND	—	—	CR
2	F/3	2.0	Abdomen	336	Normal	Imbalance	—	—	CR
2	F/10	2.0	Abdomen	484	Gain	Normal	24.0	Local	DPD
2	F/6	3.3	Abdomen	949	Normal	Normal	15.0	Local	CR
									
2	F/43	Negative	Abdomen	NE	Normal	Deleted	30.0	Local	CR
2	M/7	Negative	Abdomen	1481	Normal	ND	3.8	Local	CR
2	F/11	Negative	Neck	444	Normal	Imbalance	25.1	Local	AWD
2	M/1	Negative	Abdomen	409	Normal	Deleted	5.0	Local	DPD
3	M/15	2.0	Thorax	477	Normal	Imbalance	—	—	CR
									
3	F/50	4.5	Abdomen	735	Normal	Deleted	—	—	CR
3	F/15	5.0	Abdomen	1051	Normal	Normal	—	—	CR
3	F/6	20.0	Abdomen	791	Normal	Normal	—	—	CR
3	F/8	34.6	Abdomen	437	Normal	Normal	8.1	Local	CR
3	F/31	29.1	Abdomen	2445	Amplified	ND	13.3	Local	DPD
3	F/18	155.0	Abdomen	ND	Normal	Normal	1	Local	DPD
3	M/73	1.0	Thor/Abd	800	ND	Imbalance	13.2	Local	CR
									
3	F/11	Negative	Abdomen	6247	Amplified	Deleted	7.0	Local	DPD
3	F/118	Negative	Abdomen	127	ND	Imbalance	7.0	Local	AWD
3	M/17	Negative	Abdomen	4672	Amplified	Deleted	1.0	Local	DPD
3	M/13	Negative	Neck	536	Amplified	Deleted	14.0	Local	DPD
3	M/7	Negative	Abdomen	401	Normal	Deleted	17.0	Local	CR

a AWD=alive with disease; CR=complete remission; DPD=dead of progressive disease; F=female; M=male; ND=not determined.

**Table 3:** EFS of patients with localised NB stratified according to the indicated variables

**Variables**	***n***	**5-year EFS %**	**s.e.^a^**	***P***
*GD2*				<0.001
Negative	126	89.9	0.03	
Positive	19	62.2	0.36	
				
*Gender*				0.166
Male	70	90.5	0.03	
Female	75	81.2	0.05	
				
*Age*				0.352
<12 months	70	83.6	0.05	
⩾12 months	75	88.7	0.04	
				
*Primary site* b				0.211
Abdomen	105	84.1	0.04	
Thorax	40	91.6	0.05	
				
*Tumour stage (INSS)*				0.033
Stages 1–2	105	89.8	0.03	
Stage 3	40	76.7	0.07	
				
*NSE (78 tested)*				0.445
<100 ng ml^−1^	69	86.1	0.04	
⩾100 ng ml^−1^	9	77.8	0.14	
				
*LDH (123 tested)*				0.010
<1000 IU ml^−1^	101	89.5	0.03	
⩾1000 IU ml^−1^	22	72.1	0.10	
				
*Ferritin (95 tested)*				0.604
<150 ng ml^−1^	74	84.4	0.04	
⩾150 ng ml^−1^	21	80.0	0.09	
				
*Myc-N (124 tested)*				<0.001
Not amplified	110	90.4	0.03	
Amplified	14	57.1	0.13	
				<0.001
*1p36 (105 tested)*				
Not deleted	71	92.7	0.03	
Deleted	16	45.8	0.14	
Imbalance	18	83.0	0.09	

EFS=event-free survival; NB=neuroblastoma.

a Standard error.

b Abdomen=adrenal+other abdominal site; thorax=thorax+neck.
