# Supplementary material for: Detection of GD2-positive cells in bone marrow samples and survival of patients with localised neuroblastoma
Source: Br J Cancer. 2008 Jan 8;98(2):263–9. doi: 10.1038/sj.bjc.6604179 (PMC2361437; doi:10.1038/sj.bjc.6604179)
Supplement: Supplementary data [file 6604179x1.doc]

Supplemental data to the manuscript

“Detection of GD2 positive cells in bone marrow samples and survival of patients with localized neuroblastoma” by Corrias et al.

Table 1. Characteristics of patients diagnosed with localised NB during the study period and stratified on availability of GD2 status at diagnosis.

| Characteristic | GD2 info available | | *P* |
| --- | --- | --- | --- |
|  | Yes (n=145) | No (n=195) |  |
| Sex, male (%) | 75 (51.7) | 98 (50.3) | *0.789* |
| Age at dx (months: median, IQR) | 13 (3-36) | 9 (2-29) | *0.231* |
| Stage n,(%)  1  2  3 | 66 (45.5)  39 (26.9)  40 (27.6) | 85 (43.6)  43 (22.1)  67 (34.4) | *0.351* |
| *Myc-N* amplified n/tested, (%) | 14/124 (11.3) | 10/173 (5.8) | *0.086* |
| 5 y EFS% (se) | 86.2 (3.0) | 85.0 (2.9) | *0.959* |
| 5 y OS% (se) | 92.9 (2.4) | 91.4 (2.4) | *0.809* |

#### Table 2. Relative risk of relapse or death in GD2 positive versus GD2 negative patients, estimated by the Hazard Ratio (HR) in multivariable Cox regression models.

|  | Risk of relapse | | Risk of death | |
| --- | --- | --- | --- | --- |
| Risk Factor | HR | 95%CI | HR | 95%CI |
| GD2 |  |  |  |  |
| Negative | 1 (ref) | - | 1 (ref) | - |
| Positive | 4.9 | 1.5-15.6 | 4.5 | 1.0-20.1 |
| *Myc-N* |  |  |  |  |
| Not amplified | 1 (ref) | - | 1 (ref) | - |
| Amplified | 2.9 | 0.76-11.5 | 7.0 | 1.1-45.6 |
| 1p36 |  |  |  |  |
| Not deleted | 1 (ref) | - | 1 (ref) | - |
| Deleted | 7.0 | 2.0-24.3 | 8.4 | 1.2-58.9 |
| Stage |  |  |  |  |
| 1-2 | 1 (ref) | - | 1 (ref) | - |
| 3 | 0.73 | 0.19-2.8 | 0.71 | 0.15-3.4 |
| LDH |  |  |  |  |
| < 1000 IU/ml | 1 (ref) | - | 1 (ref) | - |
| ≥ 1000 IU/ml | 1.3 | 0.20-8.4 | 1.7 | 0.19-14.2 |

Note: 1p36 imbalance was included in the “not deleted” category. HRs for GD2, *Myc-N* and 1p36 status were estimated after having excluded the other two predictors (*i.e.*, LDH and Stage) in order to obtain more stable estimates.
